# Mapping global research on climate and health using machine learning (a systematic evidence map)

**DOI:** 10.12688/wellcomeopenres.16415.1

**Published:** 2021-01-20

**Authors:** Lea Berrang-Ford, Anne J. Sietsma, Max Callaghan, Ja C. Minx, Pauline Scheelbeek, Neal R. Haddaway, Andy Haines, Kristine Belesova, Alan D. Dangour

**Affiliations:** 1 Priestley International Centre for Climate, University of Leeds, Leeds, LS2 9JT, UK; 2 Mercator Research Institute on Global Commons and Climate Change, Torgauer Straße 12–15, EUREF Campus #19, Berlin, 10829, Germany; 3 Centre on Climate Change and Planetary Health, London School of Hygiene & Tropical Medicine, Keppel Street, London, WC1E 7HT, UK; 4 Stockholm Environment Institute, Linnégatan 87D, Stockholm, Sweden; 5 Africa Centre for Evidence, University of Johannesburg, Johannesburg, South Africa

**Keywords:** Climate, health, mitigation, adaptation, global, machine learning, topic modelling, systematic

## Abstract

Climate change is already affecting health in populations around the world, threatening to undermine the past 50 years of global gains in public health. Health is not only affected by climate change via many causal pathways, but also by the emissions that drive climate change and their co-pollutants. Yet there has been relatively limited synthesis of key insights and trends at a global scale across fragmented disciplines. Compounding this, an exponentially increasing literature means that conventional evidence synthesis methods are no longer sufficient or feasible. Here, we outline a protocol using machine learning approaches to systematically synthesize global evidence on the relationship between climate change, climate variability, and weather (CCVW) and human health. We will use supervised machine learning to screen over 300,000 scientific articles, combining terms related to CCVW and human health. Our inclusion criteria comprise articles published between 2013 and 2020 that focus on empirical assessment of: CCVW impacts on human health or health-related outcomes or health systems; relate to the health impacts of mitigation strategies; or focus on adaptation strategies to the health impacts of climate change. We will use supervised machine learning (topic modeling) to categorize included articles as relevant to impacts, mitigation, and/or adaptation, and extract geographical location of studies. Unsupervised machine learning using topic modeling will be used to identify and map key topics in the literature on climate and health, with outputs including evidence heat maps, geographic maps, and narrative synthesis of trends in climate-health publishing. To our knowledge, this will represent the first comprehensive, semi-automated, systematic evidence synthesis of the scientific literature on climate and health.

## Introduction

There are a range of causal pathways linking climate change to the environmental and social determinants of health ^1^. Climate change is already affecting local and regional weather patterns, with implications for the frequency and intensity of extreme events such as heatwaves, flooding, drought, and wildfires ^2– 5^. Increased ambient temperatures, that have been observed in nearly all geographic regions, have a direct effect on public health ^6^. More complex pathways of impact include those mediated through ecosystems such as effects on nutritional health through food systems: changing temperature and rainfall already affect crop yields ^7– 10^. Other effects on health are mediated through socioeconomic pathways including increasing poverty and migration ^11– 14^. Overall, climate change is likely to disproportionately affect the poorest countries.

The scientific literature base on climate change and health relationships is large and fast-growing, making systematic assessment of the breadth of evidence difficult using conventional, largely manual methods. A search in Web of Science for documents with “climat*” and “health*’ within title-abstract-keywords, for example, retrieves >35,000 documents published in the past five years alone. Expanding this search to include weather or extreme events (e.g. floods, heat waves, drought) and specific health outcomes (e.g. malaria, nutritional deficiencies, diarrhoeal illness) rapidly increases the number of retrieved documents to over 200,000. For example, Bouzid *et al.* ^15^ systematically reviewed systematic reviews on the effectiveness of public health interventions to reduce the health impact of climate change, synthesising literature up to 2010. Their review retrieved and screened 3,176 records, including 33 in the review. Replication of their search strategy now indicates that there have been >10,000 new scientific publications since 2010, and their publication notably excluded single studies, health-relevant interventions outside the health sector, and a range of terms relevant to health such as food security, mental health, and chronic disease.

This literature ‘explosion’ ^16, 17^ has meant that delivering transparent, systematic, and robust evidence synthesis is increasingly difficult, and evidence maps that use traditional approaches are increasingly relying on a smaller and smaller portion of the literature to inform policy and practice for climate adaptation and resilience ^16, 18^. New ‘big data’ tools have recently become available that allow us to scale evidence synthesis to potentially vast literatures ^19, 20^. Rather than increasingly restricting the scope of reviews, these tools herald a new era of large-scale computer-assisted evidence synthesis (e.g. 17, 18, 21, 22). This protocol outlines the methods used to conduct a machine learning-assisted systematic evidence synthesis of the global literature on climate and health. We are guided by methods for systematic mapping, which are adapted here for the context of machine learning as applied to large-n literature.

## Stakeholder engagement

The overarching research questions and broad scope of the work were outlined by the UK Foreign, Commonwealth and Development Office (FCDO). This protocol was developed and refined in partnership with FCDO and experts at the Priestly International Centre for Climate, University of Leeds, and the Centre on Climate Change and Planetary Health at LSHTM. In addition, an independent review panel, constituted to provide expert external oversight, contributed detailed advice and feedback on the draft protocol. The independent panel comprised:

-  Professor Kristie Ebi, Centre for Health and the Global Environment, University of Washington, USA;

-  Professor Howard Frumkin, School of Public Health, University of Washington, USA;

-  Dr. Shuaib Lwasa, Global Centre on Adaptation, Netherlands (formerly Makerere University, Kampala, Uganda)

-  Dr. Chandni Singh, School of Environment and Sustainability, Indian Institute for Human Settlements, Bangalore, India.

## Objectives

Our primary objective is to systematically synthesize the global evidence on the relationship between climate change, climate variability, and weather (CCVW) and human health. We framed our review using standards for formulating research questions and searches in systematic reviews, using a PICoST approach: population/problem (P), interest (I), context (Co), and time and scope (T/S) ( Table 1).

**Table 1.** Review objectives and key elements.

**Review** **objective(s)**	To systematically synthesize the evidence on the relationship between climate change, climate variability, and weather (CCVW) and health globally
**Population(P)**	Global, human
**Interest (I)**	Empirical evidence on the relationship between climate change, climate variability, and weather (CCVW) and human health
**Context (Co)**	Any component of the nexus between climate change, climate variability, and weather (CCVW) and human health, including impacts on health, and responses to reduce health impacts from climate change (e.g. adaptation, mitigation), without prejudice to any climate-health pathways
**Time & Scope** **(T/S)**	Scientific articles and reviews published between 2013 and 2020

### Scope and key concepts

**Figure 1.**
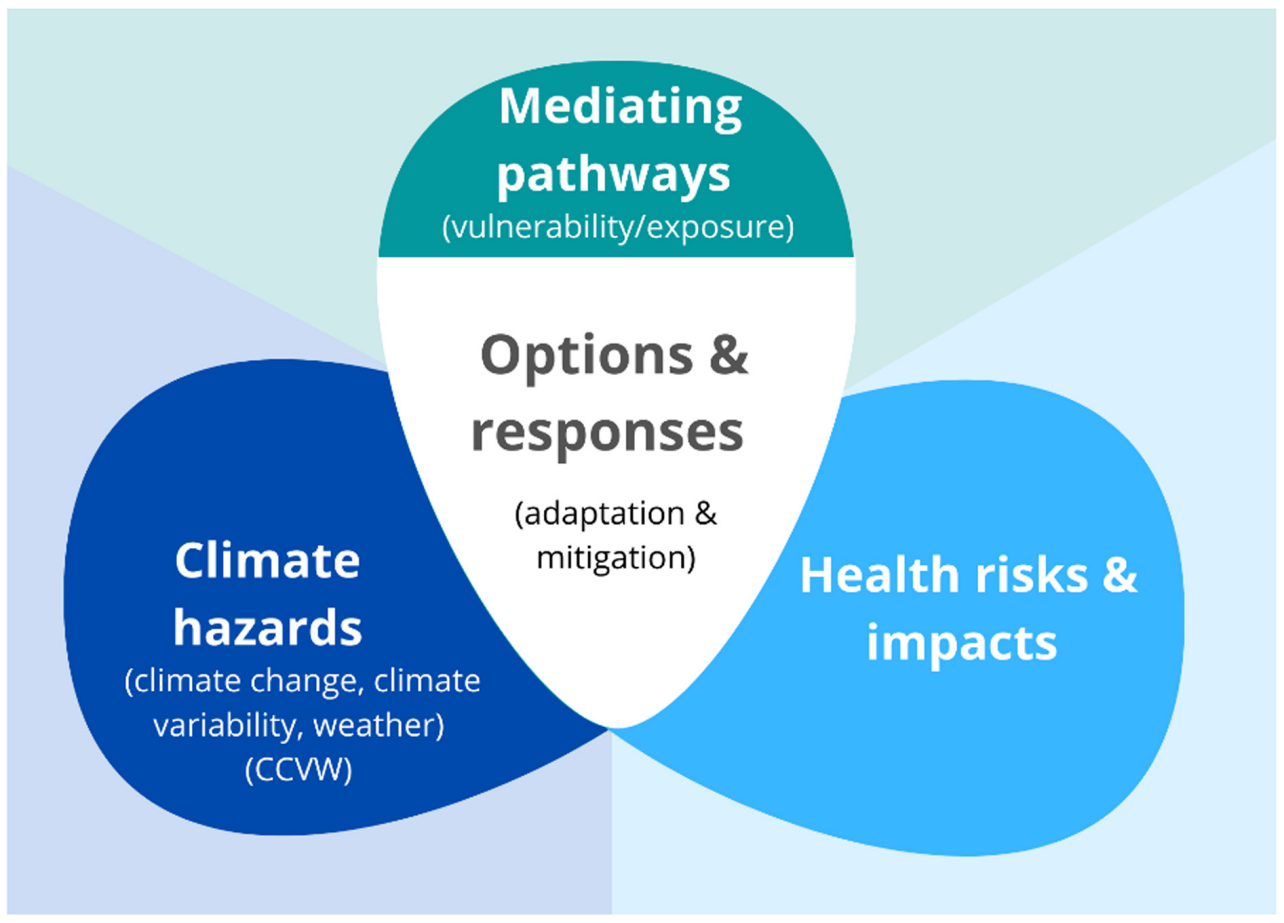
Conceptual framework and key concepts.

This systematic evidence synthesis protocol uses a framework and definitions adapted from the Intergovernmental Panel on Climate Change (IPCC)’s ( Figure 1) and we use the most recent IPCC definitions ^23^, adapted to the context of health. We include key climate hazards, health impacts and risks, mediating pathways, and adaptation/mitigation options and responses ( Figure 1).

**Climate hazards** are defined as changes to global climate and their impacts on meteorological variability and climate-related events. We collectively refer to climate change, climate variability, and weather (CCVW) to encompass trends in climate that can be attributed to climate change and weather-related impacts that change in frequency and/or intensity due to climate change. We also include natural regional climate phenomena, notably El Nino, that provide analogues of rapid climate change.

**Health risks** (potential for adverse health consequences) **and impacts** (consequences or outcomes of realised risks) include the wide-ranging health outcomes that are affected by climate change via diverse and often complex causal pathways. We include health outcomes as well as the proximal determinants of health outcomes, including air quality, vector habitat, food security, water, sanitation and hygiene, and health systems.

**Options and responses** include the range of human strategies and measures that can be deployed to minimize the negative health impacts of climate change. These can be in response to real, anticipated, or perceived climate risks and impacts, and may be reactive or proactive. *Mitigation* responds directly to climate drivers by aiming to reduce greenhouse gas emissions, and therefore focuses intervention on reducing the magnitude of climate hazards as well as capitalising on co-benefits of mitigation (e.g. from reduced air pollution). *Adaptation* aims to minimise the impact of climate hazards on humans and ecosystems of importance to humans by focusing intervention on the pathways that mediate climate impacts (i.e. by reducing vulnerability and exposure).

**Mediating pathways** determine the magnitude and nature of the effects of climate change on health, and reflect the ways in which climate hazards indirectly impact health via effects on the existing determinants of health. The IPCC refers to these as exposure and vulnerability ^23^. We collectively define exposure and vulnerability as mediating pathways to align more closely with how these components are understood within a health context. We consider mediating pathways to comprise the role of non-climatic factors in mediating, effect modifying, and interacting with climate hazards to influence health risks and outcomes. Exposure reflects the ways in which humans are located and live in places and spaces that put them at greater risk of impact, such as the location and development of infrastructure or social and cultural assets in high-risk areas or settings. Vulnerability is defined by the IPCC as the propensity or predisposition to be adversely affected, and encompasses a range of factors affecting sensitivity or susceptibility to harm and lack of capacity to cope and adapt ^23^. Vulnerability describes the social, cultural, economic, and demographic factors that mediate how hazards will manifest as impacts.

## Methods

The methodology proposed in this protocol is guided by principles of systematic mapping ^24^ and the protocol conforms to ROSES reporting standards ^25^. Forms for ROSES systematic map protocols are included in *Extended data* (SM2) ^26^.

### Search strategy

*Information sources*: The search will be carried out on three different databases: Web of Science Core Collection ^1^, Scopus, and Medline ( Table 2). The former two are general scientific databases whereas the latter is aimed primarily at biomedical and life sciences, including public health. For all databases except Web of Science Core Collections, the search will be carried out on title, abstract and keywords. Searches using keywords in Web of Science automatically generate additional default searches (referred to as “keywords plus”), which act to introduce a huge volume of irrelevant literature and expand the specificity -- and reduce the transparency -- of our search string. We will thus limit searches in Web of Science to title and abstract only. Results will be de-duplicated using trigram similarity on titles, combined with having a publication year within one year of each other or matching at least one author surname. 

**Table 2.** Overview of database search parameters and number of documents retrieved per database.

Database	Search on	Estimated N. of documents ^*=*^	Limiting parameters
**Web of Science** **Core Collection**	Title or abstract	Approx. 175,000	Document type (article or review only -- excluding book chapters, proceedings, comments, and editorials), publishing year, some excluded journals.
**Scopus**	Title, abstract or keywords	Approx. 300,000	
**Medline**		Approx. 50,000	
**Total (after** **duplicate removal)**		Approx. 350,000	

Many relevant search terms result in a high rate of irrelevant documents. To increase relevance of our search without removing relevant terms that could not be reasonably specified further, we will exclude search results from journals with a high rate of retrieval of irrelevant publications ^2^. This journal exclusion list is restricted to highly-focused titles in material sciences, physics, and chemistry ( Table 3).

**Table 3.** Journals excluded from searches.

ACS Applied Materials And Interfaces ACS Nano Analytical Chemistry Biomacromolecules Bioresource Technology BMC Genomics Carbohydrate Polymers Ceramics International Colloids And Surfaces B Biointerfaces Extremophiles Industrial Engineering Chemistry Research International Journal Of Biological Macromolecules International Journal of Heat and Mass Transfer International Journal Of Molecular Sciences International Journal Of Systematic And Evolutionary Microbiology Journal Of Alloys And Compounds Journal Of Applied Polymer Science Journal Of Chromatography B Journal Of Controlled Release	Journal Of Hazardous Materials Journal Of Materials Chemistry B Journal Of Molecular Liquids Journal of Nanoscience And Nanotechnology Journal Of Pharmaceutical And Biomedical Analysis Journal Of Physical Chemistry B Journal Of Physical Chemistry C Journal Of Thermal Analysis And Calorimetry's source details Journal of Thermal Biology Langmuir Materials Science And Engineering C Methods In Molecular Biology Nanoscale Palaeogeography Palaeoclimatology Palaeoecology Physical Review B Resuscitation RSC advances Sensors And Actuators B Chemical

### Search string

Our search string combines two key concepts: climate change and health. We first developed a set of initial search terms from existing keystone articles and reviews, assessment of IPCC reports, and internal team expertise. This step ensured that our search strings did not exclude common terms used in the literature. We subsequently tested alternate combinations of search terms iteratively to compare results from different databases and identify further missing areas, as well as terms that reduced precision (e.g. high rate of irrelevant hits). Strings were designed to minimise inclusion bias induced by the search: the focus on inclusivity allows the search strings to comprehensively identify lesser known or understudied pathways that may have received less attention in previous reviews and reports. Search strings were reviewed by all team members, as well as external expert advisors, and revised accordingly.

Consistent with the scope of our review, our climate-related search string includes terms reflecting climatic and meteorological processes, as well as vocabulary reflecting impacts, adaptation, and mitigation, but excluding intermediate pathway terms. Selection of terms is based on key vocabulary articulated in the Intergovernmental Panel on Climate Change’s 5th Assessment Report and Special Report on Global Warming of 1.5°C ^23, 27^. Adaptation is not included as the term in the final search string since it is a highly generalized term associated with a large number of irrelevant topics; and all relevant literature using the term is assumed to include reference to ‘climate’.

Development of the search string for health is similarly aligned with the stated scope, and focused on health outcomes. While we explicitly include literature relevant to health policy and systems, our scoping phase identified no additional search terms needed for this literature, which is captured under our use of general search terms (e.g. ‘health*’). We include all pathways linking climate change to human health, and focus our search strings on retrieving literature linking climate change and climate variability to human health outcomes, and literature linking meteorological processes to human health outcomes. This does not exclude any causal pathways from the searches, but rather increases the specificity of searches to exclude irrelevant literature that does not consider climate/meteorological change and variability, or is not relevant to health.

All searches use English-only search terms. Bibliographic databases include articles in all languages indexed with English translations of title, keywords, and abstract. We do not restrict by language in our inclusion and exclusion criteria.

Search strings were reviewed, discussed, tested, and finalised across the team. The resulting search strings for the climate and the health components can be found in Table 4.

**Table 4.** Summary of search strings. Number of hits based on a preliminary search. Each of the strings is connected by a boolean ‘OR’. The Scopus search string is given here; for Web of Science and Medline, the syntax is different, and some other minor changes were made, most notably removing left-truncated keywords. Search hits shown in the table were conducted on 9 April 2020. Note the following data search functions: * = any subsequent letters; W/# = maximum number of words allowed between the term directly to the left and that directly to the right of the W/#; and ? = any letter or space to replace the “?”.

Theme	Key concepts	String (Scopus)	Attributable Hits (scopus)
**Climate change** *(contains at* *least one of the* *following climate* *terms, from any* *category)*	General climate change terms	(climat* OR "global warming" OR "greenhouse effect*")	35,052
	Greenhouse gasses, including short-lived greenhouse gasses, when linked to emission or mitigation. Some astronomy results are filtered out.	(("carbon dioxide" OR co2 OR methane OR ch4 OR "nitrous oxide" OR n2o OR "nitric oxide" OR "nitrogen dioxide" OR nox OR *chlorofluorocarbon* OR *cfc* OR refrigerant OR hydrofluorocarbon* OR hfc* OR *chlorocarbon* OR "carbon tetrachloride" OR ccl4 OR halogen* OR ozone OR o3 OR ammonia OR nh3 OR "carbon monoxide" OR co OR "volatile organic compounds" OR nmvoc OR "hydroxyl radical" OR "oh" OR "pm2.5" OR aerosol OR "black carbon" OR "organic carbon" OR "sulphur dioxide" OR "oxidized sulphur" OR "so2" OR "sox" OR "sulphuric acid" OR so4* ) W/2 (emit* OR emission OR releas* OR mitigat*) AND NOT(star OR "solar system"))	7,871
	Climate variability indicators/climate indices	(temperature* OR precipitat* OR rainfall OR "heat ind*" OR "extreme-heat event*" OR "heat-wave" OR "extreme-cold*" OR "cold ind*" OR humidity OR drought* OR hydroclim* OR monsoon OR "el ni$o" OR enso OR SOI OR "sea surface temperature*" OR sst)	199,558
	Complex climate indices, including extreme weather events, floods, wildfire, and coastal changes. Some paleo-climatic events are excluded.	(snowmelt* OR flood* OR storm* OR cyclone* OR hurricane* OR typhoon* OR "sea-level" OR wildfire* OR "wild-fire*" OR "forest-fire*" OR ( ( extreme W/1 event* ) AND NOT paleo* ) OR "coast* erosion" OR "coastal change*" OR ( disaster* W/1 ( risk OR manag* OR natural)))	22,031
**AND** **Health** *(contains at* *least one of the* *following health* *terms, from any* *category)*	General health terms	(health* OR well?being OR ill OR illness OR disease* OR syndrome* OR infect* OR medical*)	49,773
	General health outcomes	(mortality OR daly OR morbidity OR injur* OR death* OR hospital* OR {a&e} OR emergency OR emergencies OR doctor OR gp)	33,571
	Nutrition, including obesity and undernutrition	(obes* OR over?weight OR under?weight OR hunger OR stunting OR wasting OR undernourish* OR undernutrition OR anthropometr* OR malnutrition OR malnour* OR anemia OR anaemia OR "micronutrient*" OR "micro?nutrient*" OR diabet*)	2,239
	Cardio-vascular terms. Some studies on Chemical Vapour Deposition (CVD) are excluded.	(hypertension OR "blood pressure" OR stroke OR *vascular OR (cvd AND NOT(vapour or vapor)) OR "heart disease" OR isch?emic OR cardio?vascular OR "heart attack*" OR coronary OR chd)	6,047
	Renal health terms	(ckd OR renal OR cancer OR kidney OR lithogenes*)	4,934
	Effects of temperature extremes	((heat W/2 (stress OR fatigue OR burn* OR stroke OR exhaustion OR cramp* ) ) OR skin OR fever* OR renal* OR rash* OR eczema* OR "thermal stress" OR hypertherm* OR hypotherm*)	23,846
	Maternal health outcomes	(pre?term OR stillbirth OR birth?weight OR lbw OR maternal OR pregnan* OR gestation* OR *eclampsia OR sepsis OR oligohydramnios OR placenta* OR haemorrhage OR hemorrhage)	2,041
	Vector-borne diseases	(malaria OR dengue* OR mosquito* OR chikungunya OR leishmaniasis OR encephalit* OR vector- borne OR pathogen OR zoonos* OR zika OR "west nile" OR onchocerciasis OR filiariasis OR lyme OR tick?borne)	2,257
	Bacterial, parasitic and viral infections, including waterborne and foodborne diseases	(waterborne OR “water borne” OR diarrhoea* OR diarrhe*l OR gastro* OR enteric OR *bacteria* OR viral OR *virus* OR parasit* OR vibrio* OR cholera OR protozoa* OR salmonella OR giardia OR shigella OR campylobacter OR food?borne OR aflatoxin OR poison* OR ciguatera OR((snake* OR adder*) W/2 bite*))	46,064
	Respiratory outcomes	(respiratory OR allerg* OR lung* OR asthma* OR bronchi* OR pulmonary* OR copd OR rhinitis OR wheez*)	3,432
	Mental health outcomes	(mental OR depress* OR *stress* OR anxi* OR ptsd OR psycho* OR *trauma* OR suicide* OR solastalgi*)	12,616
	Health systems	[no additional terms needed]	

### A machine-learning approach

Machine learning allows for the analysis of large and diverse literature bases, and involves training a computer to conduct some components of the work automatically. There are two main types: *Unsupervised machine learning* finds structures or patterns in large datasets and can be used to categorise documents. *Supervised machine learning* is based on the concept that a computer algorithm can be trained to predict the decisions that would be made by a human screener or coder. To do so, humans manually screen or code a sample of data. For evidence synthesis, a supervised machine learning algorithm is trained with human-screened articles, and can use these to predict both 1) whether an article is relevant, and 2) how the document should be categorised, based on the presence and frequency of key words used in the title and abstract. The algorithm provides a score (e.g. 0–1), predicting the likelihood that the article is relevant and/or belongs to a labeled category. Supervised machine learning requires humans to manually screen or code iterative samples of data, after which an algorithm uses this sample to classify the rest of the data. In this evidence synthesis, supervised learning will be used to predict which articles are relevant to CCVW and health, as well as to predict whether the article is relevant to adaptation, impacts or mitigation. Topic modelling, an unsupervised method, will be used in our analysis to identify key themes in the literature.

The machine learning algorithm has been developed. Relevant coding is available on GitHub ^28^. 

### Screening strategy

Given the size of the literature and the timeline of this work, not all documents can be screened by hand. At the same time, however, the broad search string and scope of this review results in a large number of irrelevant studies. To identify relevant documents within the larger set of documents retrieved by our search strings, we will use *supervised machine learning*. This approach involves manually screening (human coding) subsets of documents to iteratively ‘teach’ an automated classifier which documents are relevant according to a set of pre-defined criteria, and then use this trained classifier to predict the ‘most likely to be relevant’ literature. To be labelled as relevant, documents need to include empirical data (qualitative and/or quantitative) on both CCVW and health. All screening and analyses will be conducted on the NACSOS platform (NLP-Assisted Classification, Synthesis, and Online Screening) ^29^.

### Consistency checking

A sample (>10%) of screened documents will be reviewed by multiple team members; the documents in these samples that are labelled differently by different team members will be discussed until consensus is reached, to reduce bias and ensure consistency between team members. We will not consult full text during this process.

It is common practice to divide the manually classified set into a training set and a smaller validation set. Here, we will use 10-fold cross-validation, meaning that the data will be segmented at random into 10 equal sections. A classifier is then trained on 90% of the screened data, providing predictions for the remaining 10%, after which the training is repeated withholding a different section for validation. The result is a relevance prediction for all screened documents, based on an algorithm trained with 90% of the screened data. The results of the classifier on the different validation sets thus includes both false positives (i.e. the algorithm included the article, but the human reviewer did not) and false negatives (i.e. the inverse). If a certain kind of document occurs often in either one of these error categories, this could point to inconsistencies in the manual coding and re-assessing the documents in these error categories can help improve the accuracy of the classifier as well as uncover ambiguities in the screening protocol. To find these inconsistencies, the false positives and false negatives from the validation sets will be re-assigned for screening to see if the initial human label was correct. In essence, this allows the reviewers to use a preliminary version of the algorithm as an extra consistency check. Inclusion and exclusion criteria are summarized in Table 5.

**Table 5.** Screening and tagging criteria for supervised machine learning. For a document to be included, it must meet all inclusion criteria for at least one tag. Tags are not mutually exclusive. Details of inclusion and exclusion screening and tagging criteria are provided in the *Extended data* (SM1) ^26^.

Inclusion	Exclusion	Tag (for included articles only)
Includes substantial focus and empirical data (qualitative or quantitative) or secondary analysis of data on a climate-related driver of impacts, AND Includes substantial focus and empirical data (qualitative or quantitative) or secondary analysis of data on a perceived ^1^, experienced, or observed eligible ^2^ health-related outcome or health system	Does not include an eligible climate-related driver of impacts, or a health-related outcome or health system. OR Does not include empirical data for both of the above. OR Consideration of climate drivers and/or health outcomes is a minor or tangential component of the document	Impacts
OR		
Includes empirical data (qualitative or quantitative) or secondary analysis of a driver of climate change, OR secondary analysis of a mitigation or energy production/efficiency measure AND Includes substantial focus or consideration of a perceived, experienced, or observed impact on an eligible health-related outcome or health system	Reference to a driver of climate change, mitigation, energy production or efficiency is not accompanied by relevant empirical data and/or analysis. OR There is no reference to impacts on eligible health-related outcomes or health systems OR The document focuses on emissions within the healthcare sector with no consideration of impacts on health.	Mitigation
OR		
Includes substantial focus (documenting and/or empirically assesses) a human response ( adaptation) to perceived, experienced, or observed impacts on eligible health-related outcome or health system	Document focuses on potential or planned responses to the health impacts of climate change. OR Responses (adaptations) are not relevant to human health (e.g. conservation measures) OR Responses (adaptations) that are not clearly linked to eligible health-related outcomes or health systems (e.g. general resilience activities)	Adaptation

1 Perceived impacts are based on the perspective of the study (authors and/or respondents). For example, households or governments might undertake adaptation in response to the perceived risk of flooding, regardless of whether flooding in that context has been attributed to climate change or is expected to increase under climate change projections. 2 Eligible health-related outcomes are described in the *Extended data* (SM1) ^26^.

### Inclusion criteria

*Eligibility criteria*: For all searches, the publishing year will be limited to 2013–2020 to capture literature published since the last IPCC Assessment Report. We further limit our review to published research papers and reviews only. We frame our review using standards for formulating research questions and searches in systematic reviews, using a PICoST approach: population/problem (P), interest (I), context (Co), and time and scope (T/S) ( Table 1). The review population (P) included all nations globally. The focus of interest (I) was the relationship between CCVW and health, specifically, the evidence base for the different components of this relationship. The time period (T) is 2013 to 2020. Screening will focus on identification of documents that meet PICoST search criteria. This means that documents must be indexed in English and:

1. Provide a clear link to actual, projected, or perceived impacts of climate change, responses to reduce the impacts of climate change (adaptation), or the mitigation of greenhouse gas emissions. Evidence of detection and attribution was not required.
2. Include substantial focus on a perceived, experienced, or observed eligible health-related outcome or health system.
3. Present empirically-driven research or a review (including non-systematic reviews) of such research.

### Critical appraisal

Our evidence synthesis process is guided by systematic mapping methods, and as such does not include critical appraisal of study validity nor full synthesis, but rather describes the nature of the evidence base. Furthermore, the evidence synthesis is supported by machine learning practices to reduce workload and optimise timeliness and resource efficiency.

### Data extraction

We will extract the bibliographic meta-data for all documents retrieved through search strings from bibliographic databases. Documents where the abstract is missing will be removed from the dataset as these abstracts are crucial for the inclusion/exclusion classifier. If any of the other data fields is missing, the document will still be included in the dataset. All data extraction activities will be conducted using abstracts and titles only. No data extraction will be conducted using full texts.

*Climate categories:* Supervised machine learning will be used to categorize documents into three climate literature categories: adaptation, mitigation, and climate impacts ( Table 5). This data extraction will be undertaken in parallel with the screening described above. A sample of the articles will be manually tagged (identical sample to that used for screening), and an algorithm will be used to predict the likely relevance of the remaining articles to each climate category. All documents are assumed to fall into at least one of the three categories in order to have met our original inclusion criteria. Documents can be tagged as relevant to more than one category (e.g. impacts AND adaptation).

*Geographical location:* We will use ‘geoparsers’ to classify documents based on their geographic focus. Geoparsers refer to algorithms that can extract geographic place names from text, based on dictionary methods or pre-trained models. We will employ a geoparser to determine the country of affiliation for the first author of the paper, as well as to identify which countries or places within countries are mentioned in abstracts. When author information is missing, the geoparser cannot determine the affiliation of the author, but it should be able to provide an estimate for nearly every case where author information is given. Similarly, a substantial number of abstracts will not contain any geographic place names as many studies either are place-independent or do not mention the specific case they are investigating in the abstract. For these articles, geographical data cannot be collected.

*Thematic topics:* We will use machine-learning approaches – in this case an *unsupervised machine learning* approach called *topic modelling* – to identify thematic topics in the included articles. Topic modelling is a method that automatically identifies clusters of words which frequently occur together. These clusters are used to assign ‘topic scores’ to each document. Topic modelling identifies a pre-specified number of topics. The themes resulting from topic modelling are not based on any labelling or tagging, but rather based on structures that the algorithm finds in the data itself. In practice, this means that words which are frequently used in different abstracts will form a topic, and that each abstract will be assigned a ‘topic score’. This score represents which words associated with a certain topic are used in that abstract. Topic modelling requires the user to *a priori* set the number of topics, as well as some hyper-parameters. To find the most relevant and interpretable topic model, we will run several topic models using both Latent Dirichlet Allocation (LDA) and Non-negative Matrix Factorization (NMF) with different parameter settings (50-80 topics in increments of five; for LDA, alpha was set at 50/[number of topics]). We will identify the number of topics providing the best balance between detail and interpretability. Topics will be assigned to one of five aggregated ‘meta-topics’ based on our review framework, including: climate hazards (CCVW), health risks and impacts, options and responses, mediating pathways, or ‘other’ ( Figure 1). In some cases, conceptually similar topics may need to be combined for ease of understanding. Expert assessment will be used to iteratively review, label and create aggregated topics to support synthesis.

### Validation of machine-learning methods

Validation of results is based on performance scores to assess how reliable a supervised machine learning method is. Accuracy describes the proportion of all documents that are correctly classified. Precision reflects the proportion of the documents labelled relevant by the algorithm that are actually relevant. Recall describes the proportion of relevant documents that are classified by the algorithm as relevant, and is analogous to diagnostic sensitivity. In practice, there is a trade-off between precision and recall: if a classifier retrieves many documents (high recall) it will likely also retrieve more irrelevant documents (false positives; low precision) and vice versa. >90% accuracy is a common performance goal but may not always be achievable or reasonable. Conceptual complexity may lead to inconsistent coding, which would in turn lead to inconsistent performance of the classifier. In other words, the performance of the classifier tends to be lowest where human classification is hardest. Our performance goal in this review is to maximize performance scores until no longer possible given the complexity of the topic. We will assess this by calculating the classifier performance for increasing sizes of the dataset, and finding the point at which performance has clearly levelled off -- i.e. more data no longer leads to better performance.

### Data synthesis

*Topic maps:* We will generate topic maps based on the outcome of the topic model. Topics maps use machine learning methods, specifically text mining, to identify groups or clusters of words that occur in a group of documents ^17^. Topics mapping is used to identify sets of words (referred to as ‘topics) that co-occur. We might find, for example, that one topic includes the words ‘agriculture’, ‘farming’, ‘crops’, and ‘drought’, reflecting a topic themed around drought risks to agriculture, while another topic includes the words ‘malaria’, ‘vectorborne’, ‘parasite’, and ‘transmission’, collectively reflecting a topic on infectious disease (specifically malaria) transmission. The labels or names given to these topics are not automated, and must be defined by the research team. Topic mapping can be used to generate a visual ‘topography’ of key topics, with frequently co-occurring topics visually located closer together than those that rarely co-occur. We will use the t-SNE dimensionality reduction algorithm to plot the topic scores for each document, labelling clusters of documents based on locally dominant topics, and overlaying the map with meta-data information. In simpler terms, we will make use of the fact that every document will have its own topic score, and each document can contain multiple topics to identify clusters of documents with similar topics. This then allows us to plot similar documents closely together. The resulting clusters are indications of conceptual linkages between topics and can give further insights into the significance of the identified topics.

*Evidence heat maps*: Heat maps will be created to visualize the relative co-occurrence of topics. Co-occurrence here will be defined as both topics constituting at least 0.15 of the topic distribution of the document.

*Evidence atlases (i.e. cartographic maps)* will be generated to visualize the geographic locations of studies.

*Narrative synthesis*: We will appraise the frequency of key topics within the climate and health literature, as well as the extent of co-occurrence of topics within the topic and heat maps. We will assess the extent to which trends in the literature differ by country income class.

### Knowledge gap and cluster identification strategy

This review approach, using machine learning to synthesize a large literature base, is explicitly designed to facilitate knowledge gap and cluster identification. Topic maps and heat maps in particular aim to visualize areas of knowledge clusters, and highlight gaps in literature for particular combinations of topics (e.g. particular heat outcomes, climate hazards, or response types).

### Demonstrating procedural independence

Given the nature of the review, reviewers will not be in a position to make primary decisions (i.e. whether an article is eligible for inclusion) on their own work. Manual coding will be conducted for a sample of the literature by an early career researcher, with team double-coding and validation on selected samples to ensure consistency. Where these validation samples include authors from the review team, decisions will be made collectively by several team members, reducing the potential for bias by a team member author. Following manual training, all inclusion decisions will be made automatically by the machine learning algorithm, reflecting robust procedural independence.

### Limitations and potential bias

There are a number of potential sources of publication bias affecting this review. The preponderance of positive and significant results in the non-grey literature means that issues where the direct causal link is clear and/or easy to quantify may be over-represented (e.g. excess mortality due to an extreme weather event has both a clear cause and a concrete proxy for health, whereas mental health effects of droughts through reduced livelihood opportunities may be harder to quantify and therefore less likely to be published). We further introduce bias through the inclusion of publications with an English title and abstract only. Even though many databases index non-English articles, there is a dominance of English-language publications in the literature more broadly. The literature is also biased towards high income regions (lower publishing rate in lower income regions). In this context, it is typically difficult to distinguish whether absence of reporting reflects lack of substantive importance and activity, or lack of publication. Further bias is introduced by limiting the search to articles and reviews in bibliographic databases of scientific articles. Including grey literature would likely better represent institutional climate policy, for example, but at the expense of the feasibility of the assessment. This bias is roughly in the same direction as the language-based bias.

### Ethics approval

This study uses publicly available data, does not involve research using human participants, animals, or plants, and thus does not require ethics approval.

### Plans for results dissemination

We will report the results of the review in an open access international peer-reviewed journal. We will hold dissemination meetings in different settings and seek to ensure that the findings are shared widely among multiple stake-holder communities.

### Study status

The study has now been completed. A manuscript presenting final results is in submission.

## Data availability

### Underlying data

No underlying data are associated with this protocol.

### Extended data

Zenodo: Extended Materials for Protocol: Mapping global research on climate and health using machine learning (systematic protocol). http://doi.org/10.5281/zenodo.4320687 ^26^.

This project contains the following extended data in ‘WOR Protocol Extended Data.pdf’:

- SM1: Detailed screening and tagging criteria
- SM2: ROSES Systematic Mapping checklist

Data are available under the terms of the Creative Commons Attribution 4.0 International license (CC-BY 4.0).

## Software availability

Source code available from: https://github.com/AnneIsARealProgrammerNow/ClimateHealth_Wellcome

Archived source code at time of publication: https://doi.org/10.5281/zenodo.4322697 ^28^.

License: MIT license

## Notes

^1^ Web of Science Core Collection here includes: Science Citation Index Expanded (SCI-EXPANDED) --1900-present; Social Sciences Citation Index (SSCI) --1900-present; Arts & Humanities Citation Index (A&HCI) --1975-present; Conference Proceedings Citation Index- Science (CPCI-S) --1990-present; Conference Proceedings Citation Index- Social Science & Humanities (CPCI-SSH) --1990-present; Emerging Sources Citation Index (ESCI) --2015-present.

^2^ Taking the 50 most recent articles as a heuristic for the journal relevance: if none of these articles was relevant and the remit of the journal did not explicitly refer to either climate or human health, the journal was excluded.
