# Supplementary material for: Mapping global research on climate and health using machine learning (a systematic evidence map)
Source: Wellcome Open Res. 2021 Jan 20;6:7. [Version 1] doi: 10.12688/wellcomeopenres.16415.1 (PMC8889042; doi:10.12688/wellcomeopenres.16415.1)
Supplement: Peer review history

# Peer review: Reviewer response for version 1

**DOI:** 10.21956/wellcomeopenres.18061.r48500

**Published:** 2022-03-01

**Authors:** Niheer Dasandi

**Affiliations:** 1 University of Birmingham, Birmingham, UK

This article describes the protocol for the use of machine learning approaches to systematically synthesise the evidence on the relationship between climate change, climate variability, and weather (CCVW) and human health. The outputs produced through this protocol will certainly make an important contribution to our understanding of the relationship between climate change and health. The authors do a very good job of clearly explaining the different steps taken to help generate the systematic evidence synthesis on the relationship between CCVW and human health.

 I have a couple of comments on the description of the protocol, which should be treated as areas that the authors may want to consider in developing this work rather than any concerns that I have with the approach taken. The main comment I have is related to the inclusion of the different links/pathways between CCVW and human health in the evidence synthesis. As the authors note (in the ‘scope and key concept’ section), there are often diverse and complex causal pathways between CCVW and health. It’s not entirely clear that the approach taken here – specifically the focus on titles/abstracts and the search terms/strings used – will enable the authors to fully capture evidence on key pathways. More specifically, as the authors note, their search terms excluded intermediate pathway terms. My question would be whether this leads to the exclusion of studies from the synthesis that clearly provide evidence on a pathway between climate change and health. For example, a study may provide important new evidence on the health benefits of active travel (e.g. cycling/cycling infrastructure) – and not mention ‘climate change’ (or any climate search terms) in the abstract. Based on the authors’ description, I don’t think this study would meet the inclusion criteria. However, the article would clearly provide important insights on the health co-benefits of a mitigation action (switch to active travel). I am sympathetic to the argument that there is only so much a review/synthesis of the evidence can include – however, this may be an issue the authors may want to consider moving forward with this work.

 The second comment relates to the inclusion of reviews in the protocol/synthesis of the evidence (in addition to original research papers). The main benefit of including review pieces in the synthesis is that it can help to shed light on aspects of the CCVW-health relationship that have received significant attention and well-established findings. However, there are potential drawbacks. Namely, it effectively risks “double-counting” evidence on specific relationships/issues, as they will be included in this analysis as individual studies and through reviews, and so may impact findings related to the data synthesis, knowledge gaps, and cluster identification. It also may have implications for the time-period of the evidence included in the synthesis, as my understanding is that a review published in 2013 that assesses studies published between 2000 and 2012 will be included in the analysis – yet the individual studies would not be included because they are outside the time-period considered here (2013-2020). Hence, if this is the case, it might be worth the authors considering this and the implications. It may be that they have already considered this, in which case it would be worth adding some discussion of this here.

 These are fairly minor comments. Overall, I found the description of the protocol to be very clear and detailed – and as noted the outputs from implementing the protocol will make an important contribution to research and policy on the relationship between climate change and health.

Is the study design appropriate for the research question?

Yes

Is the rationale for, and objectives of, the study clearly described?

Yes

Are sufficient details of the methods provided to allow replication by others?

Yes

Are the datasets clearly presented in a useable and accessible format?

Not applicable

Reviewer Expertise:

Climate change and health; international development.

I confirm that I have read this submission and believe that I have an appropriate level of expertise to confirm that it is of an acceptable scientific standard.

# Peer review: Reviewer response for version 1

**DOI:** 10.21956/wellcomeopenres.18061.r42285

**Published:** 2022-02-07

**Authors:** John N. Lavis

**Affiliations:** 1 McMaster Health Forum and Department of Health Research Methods, Evidence and Impact, McMaster University, Hamilton, ON, Canada; 2 Africa Centre for Evidence, University of Johannesburg, Johannesburg, South Africa

This article describes a protocol for using machine learning approaches to systematically synthesize global evidence on the relationship between climate change, climate variability, and weather (CCVW) and human health. The outputs arising from the execution of the protocol can help to advance the field both substantively and (through the use of machine learning) methodologically. I have two major comments (and two minor comments).

 First, I struggled with the different descriptions of the relationships being examined. The abstract helpfully clusters them into three groups – CCVW impacts on health, mitigation strategies’ impacts on health, and adaptation strategies’ impacts on health – although the wording of relationship type 3 isn’t as clear as it could be (I surmised it’s about impacts but that wording isn’t used). The PICoST summary box is not nearly as fulsome or clear as the statement in the abstract. A later statement – “we include health outcomes as well as the proximal determinants of health outcomes, including air quality, vector habitat, food security, water, sanitation and hygiene, and health systems” – provided a big ‘aha’ for me by clarifying why the abstract and the inclusion box referred to ‘health-related outcomes *or health systems*.’ What’s not clear is 1) why health system is singled out as a proximal determinant in the abstract (can’t the others be listed too?); 2) why the PICoST summary box is much less detailed.

 Second, I struggled with why the authors weren’t being bolder by making this a living evidence map (and certainly why they’re cutting off their search in 2020) and by making available the data to others who can build on it in so many ways (e.g., by adding critical appraisals and by moving beyond topic modeling to a supervised method that would move us towards a mutually exclusive and collectively exhaustive list of strategies). These additions would move the resulting outputs from great to path-breaking.

 Much less significantly, I wondered: 1) whether the authors plan to ‘mine’ the reviews they identify for primary studies or simply present separate maps of primary studies and reviews without examining overlap; and 2) whether there’s a typo in the author list (Jan Minx, not Ja Minx).

Is the study design appropriate for the research question?

Yes

Is the rationale for, and objectives of, the study clearly described?

Yes

Are sufficient details of the methods provided to allow replication by others?

Yes

Are the datasets clearly presented in a useable and accessible format?

Not applicable

Reviewer Expertise:

Evidence synthesis, evidence support, evidence-informed policymaking, evidence-informed health systems

I confirm that I have read this submission and believe that I have an appropriate level of expertise to confirm that it is of an acceptable scientific standard, however I have significant reservations, as outlined above.

# Peer review: Reviewer response for version 1

**DOI:** 10.21956/wellcomeopenres.18061.r43366

**Published:** 2021-04-28

**Authors:** Fiona Charlson

**Affiliations:** 1 School of Public Health, The University of Queensland, Herston, Qld, Australia; 2 Institute for Health Metrics and Evaluation, University of Washington, Seattle, WA, USA

This study protocol is superbly detailed and enables replication by other researchers.

 In my experience, title and abstract reviews are not always an accurate reflection of a study's content. I wonder whether this will have an impact on the accuracy of your searches. It would be good to know whether this will be addressed throughout the machine learning process.

 It would be helpful to understand in more detail what steps will be taken to revise this protocol in accordance with your validation processes. 

 Otherwise, I have no suggestions of how to improve the proposed methods at this stage. It is an exciting piece of work and I look forward to reading how this protocol performs in a future publication.

Is the study design appropriate for the research question?

Yes

Is the rationale for, and objectives of, the study clearly described?

Yes

Are sufficient details of the methods provided to allow replication by others?

Yes

Are the datasets clearly presented in a useable and accessible format?

Yes

Reviewer Expertise:

Mental health, climate change

I confirm that I have read this submission and believe that I have an appropriate level of expertise to confirm that it is of an acceptable scientific standard.
